# Laser-Assisted Micro-Solder Bumping for Copper and Nickel–Gold Pad Finish

**DOI:** 10.3390/ma15207349

**Published:** 2022-10-20

**Authors:** Sumera Kousar, Karsten Hansen, Thomas Florian Keller

**Affiliations:** Deutsches Elektronen-Synchrotron DESY, Notkestr. 85, 22607 Hamburg, Germany

**Keywords:** micro bumping, flux-less, lead-free, SAC305, flip-chip, low-force copper bonding, formic acid, copper formate, intermetallics

## Abstract

Flip-chip bonding is a key packaging technology to achieve the smallest form factor possible. Using copper as a direct under-bump metal and performing bonding under little force and at a low temperature eliminates the processing step for the deposition of a suitable wetting metal and offers an economical solution for electronic chip packaging. In this paper, various samples with copper and nickel–gold surface finishes are used to apply an in-house solder bumping, flip-chip bonding and reflow process to exhibit the bump-bond feasibility. Native oxides are reduced using process gases, and copper surface protection and solder wetting are achieved using copper formate. Lead-free 40 µm solder balls were bumped on 80 µm copper pads and 120 µm copper pillars to demonstrate a full intermetallic Cu–Cu bond as a base study for stacking applications. Using a low-force bonding technique, various chips with different dimensions were bonded at 0.5–16 MPa, followed by a reflow step at a maximum temperature of 270 °C. Then, 30 µm solder balls are utilized to bump the samples with NiAu and Cu bond pads at 50 µm pitch. A mean shear strength of 44 MPa was obtained for the 30 µm Cu samples. To the best of our knowledge, 30 µm solder bumping directly on the copper pads by producing copper formate is a novel research contribution.

## 1. Introduction

Modern chip design processes are driven by the requirements of high speed, performance and compactness. Thus, more functionality is packed in a small piece of silicon, resulting in tiny connection pads at that finest pitches. Additionally, silicon photonics has been intensively explored for more than a decade to realize fast on-chip data transfer and meet these ever-growing data-rate demands [1,2]. From the packaging point of view, suitable and compatible assembly solutions are required to maintain the integrity of the electronic integrated circuit (EIC) and the photonic integrated circuit (PIC) with a solder-reflowable fiber assembly [3]. Both chip types are usually flip-chip bonded in a 3D integration manner [4]. Stacking multiple chips on top of each other is a key approach to achieving the smallest form factors [5]. Stacked packages rely on the flip-chip bond pads and through silicon vias (TSV) for inter-chip connections.

The semiconductor industry is transitioning towards using copper [6], which is used in redistribution layers in back-end-of-line and TSV processes and the final bond-pad material [7]. Copper can be electroplated as bumps [8] or pillars [9] for fine-pitch applications. The spacer functionality of copper pillars can be very useful for stand-off purposes in complex, 3D, integrated EIC and PIC packages. Another important facilitator towards stacking is the utilization of a complete intermetallic compound (IMC) joint to avoid reliability issues due to multiple melting during successive bonding and reflow cycles. A full IMC joint has a higher melting point and can also withstand high current stressing, thereby being more resistant against electromigration failures [10].

Copper bonding under suitable process parameters is usually challenging due to the presence of unavoidable native oxides. Various copper-bonding techniques have been reported in the literature that focus on low-temperature and low-pressure processes [11,12]. The usability of a specific bonding technique and its cost effectiveness depend on the characteristics of the mating chips, the bonding process and the production volume. The hybrid and thermo-compression techniques are two common Cu–Cu bonding approaches requiring a pollution- and oxide-free pad surface of sufficiently low roughness. Surface cleaning and protection using a self-assembled monolayer [13], non-conductive film [8], wet cleaning using sulfuric acid [14], acetic-acid swabbing [15], formic-acid (FA) vapors [16] and Ar/N_2_ plasma [17] has been reported in the literature.

Low-temperature hybrid bonding or direct bond interconnecting is a popular industrial method by which to achieve the smallest possible bond pitches [18,19,20]. Surface cleaning and preparation via chemical-mechanical polishing is required to achieve sufficient planarity for atomic-scale dielectric bonding, followed by a post-annealing process [21]. It is a wafer-to-wafer/chip-to-wafer process that is economically useful for pitches at 20 µm and below. Thermo-compression bonding usually requires very high pressure and long bonding times at elevated temperatures. This approach is not favorable for temperature- and pressure-sensitive devices or chips with optical functionalities. Therefore, further techniques are employed to lower the bonding temperatures and forces. These include deposition of passivation metals (e.g., Pd, Au, Ag) [22,23] and solder on the copper pads down to 10 µm in pitch with or without a barrier metal [24]. Solder deposition not only lowers the final bonding temperature and pressure, but also compensates inhomogeneities on the copper surface. Cu–Sn solid–liquid-interdiffusion bonding has been established to realize a full IMC joint [25]. Usually, tin is either directly deposited or followed by barrier-metal deposition (typically Ni) on the copper through an electroplating process. However, the deposition process is expensive due to the mask requirements. Hence, achieving both cost effectiveness and suitable bonding parameters simultaneously remains a challenge. In recent years, an economical, laser-assisted solder-ball bumping process has been introduced and successfully applied to copper (120 µm balls at 200 µm pitch) [26]. Solder bumping directly on the copper surface is much more challenging than on the noble metals due to formation of native oxides even at ambient temperatures, thereby requiring a surface protection and oxide prevention method.

This study is based on proven in-house processes utilizing a flux-less and lead-free laser-assisted bumping and bonding technique [27]. Here, we address upgrades with copper as a direct under-bump and pillar material, 30 µm bumps at 50 µm pitch, low-force bonding and full IMC solder joints. The laser-assisted solder jetting technique was applied to wet SAC305 (96.5 wt.% Sn, 3 wt.% Ag, 0.5 wt.% Cu) solder balls on Cu and on electroless Ni-immersion Au (ENIG), or electroless Ni, electroless Pd and immersion Au (ENEPIG) under-bump metallization (UBM). In Section 2, a detailed description of the individual samples and processes is given with special attention on the pre-treatment using a formic-acid atmosphere for Cu-surface deoxidization and subsequent prevention. A temporary tacking medium and low-force bonding are applied for modules with Cu-UBM chips and for large chips independent of UBM type. Section 3 summarizes the results obtained from shear tests, optical/X-ray inspections and electrical module tests. Optimum conditions for solder wetting on copper were derived from the lifetime of the surface protector. Different types of IMCs and their growth rates, based on the process parameters, are reported for all the samples. A comparison between Cu- and Ni-based metallization is presented, focusing on the corresponding IMCs. Reliability studies in terms of temperature cycling and high-temperature storage were performed on a copper-based sample to conclude on safe working limits for copper-based joints.

## 2. Materials and Methods

### 2.1. Samples

**Figure 1:**
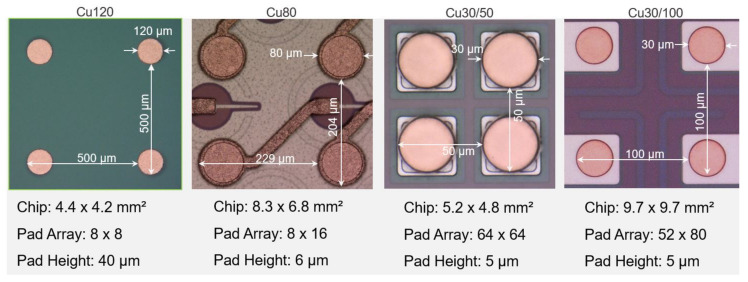
Micro photographs showing regions of four contacts of samples with Cu UBM.

**Figure 2:**
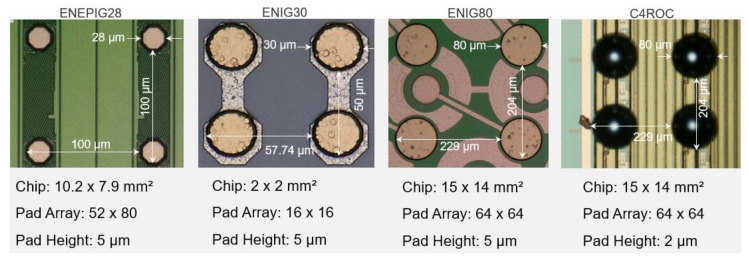
Micro photographs showing regions of four contacts of samples with Ni-based UBM.

Various samples were used to develop the process and to demonstrate the robustness and quality of the underlying bump-bond technique. Copper and Ni(Pd)Au UBM-based samples, along with their dimensions, are shown in Figure 1 and Figure 2, respectively.

Cu120 samples consisted of 40 µm high and 120 µm wide pillars. Cu120 and Cu80 samples were bonded to their identical twins to demonstrate the feasibility of bumping, low-force bonding with the help of a tacking medium and a full IMC joint. Cu30/50 samples acted as test objects for bumping on tiny pads at 50 µm pitch. Cu30/100 and ENEPIG28 samples represent sensor and readout chips of the new pixel luminosity telescope (PLT) of the CMS experiment at CERN [28]. They qualified the practicability of the full process flow for Cu UBM without a tacking medium at moderate bonding forces in a small-series production. Cu30/100 were also bonded to their identical twins to test low-force bonding and a Cu–SAC–Cu joint for 30 µm pads. ENIG30 samples were bonded to their identical counter parts and were intensively used for the optimization of solder jetting and bonding process at 50 µm pitch. ENIG80 and C4ROC samples are sensors and readout chips from the DSSC project [29]. C4ROC had 100 µm C4 bumps from the IBM and were bonded with ENIG80 and Cu80 for comparing IMCs with NiAu and Cu UBM. Due to their very large size, they had the most warpage, representing an extreme case for the low-force bonding method with a tacking medium. 30 µm and 40 µm SAC305 solder balls were used for in-house bumping.

### 2.2. Processes

**Figure 3:**
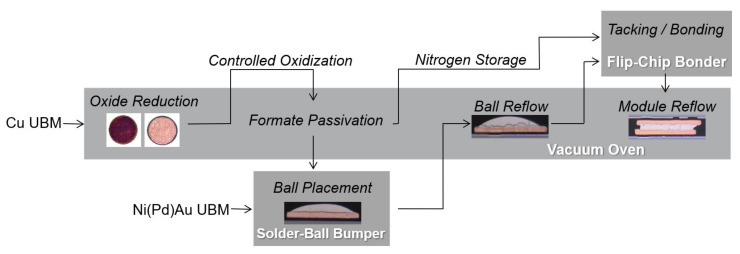
Bump-bond flow chart for chips with Ni(Pd)Au and Cu UBM. A single pad from a Cu80 sample is exemplarily shown in its initial oxidized, cleaned, bumped, ball-reflowed and final module-reflowed states for illustration.

The flow chart of our in-house bump bond process for Cu and Ni(Pd)Au metallization is summarized in Figure 3. The complete process was performed in five steps.

In the first step, the bonding surfaces were analyzed using microscopy. Since a bare copper surface is prone to oxidation, copper samples required a surface cleaning and protection before the bumping and bonding. An extreme example of an oxidized pad is shown in Figure 3. Formic acid was used to reduce the oxides and protect the surface by producing a layer of Cu formate. These reduction and protection steps were performed in a vacuum oven [30] (cf. Section 2.2.1). In the second step, one of the two mating chips was stored in the nitrogen atmosphere while the other was transferred to the solder-ball bumper [31] (cf. Section 2.2.2). In the third step, the bumped chip was transferred to the vacuum oven. Ball reflow was performed to create the characteristic spherical bump shape (cf. Section 2.2.3). In the fourth step, the reflowed chip and the one stored in a nitrogen atmosphere from step two were transferred to the flip-chip bonder for tacking [32] (cf. Section 2.2.4). The purpose of this step is to align the mating chips and hold them together for transport using moderate pressures and temperatures below melting point. The solder alloy softens and deforms at the chosen parameters without assisting further intermetallic reactions between the solder and the UBM surfaces. This step was also performed at the lowest pressures and temperatures by using an additional tacking medium to allow a low-force bonding technique. In the last step, the tacked module was transferred into the vacuum oven to achieve the final connectivity by performing reflow above the solder melting temperature (cf. Section 2.2.5).

It should be noted that the semi-closed chamber of the flip-chip bonder is not sufficient to process copper surfaces at elevated temperatures due to the oxidation issues. However, in case of Ni-based UBM, the pre- and post-bonding reflow steps can also be performed in the flip-chip bonder [27]. Moreover, Ni-based UBM with clean and noble surface finishing do not need to go through the cleaning processes but can directly be moved into the solder-ball bumper, as illustrated in Figure 3.

#### 2.2.1. Oxide Reduction and Format Passivation

**Figure 4:**
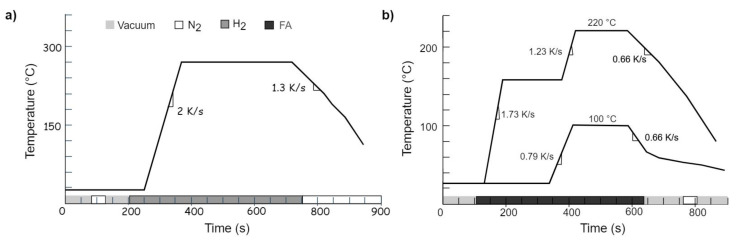
Temperature profiles for reducing Cu oxides using (**a**) hydrogen gas at 270 °C and (**b**) formic-acid vapor at 220 °C. The lower curve in (**b**) labeled with 100 °C represents the production of Cu formate.

The vacuum oven is equipped with process gas lines and a plasma source for surface treatments and oxide reduction. We tested both hydrogen and formic acid, to study the reduction efficiency for the samples with different oxide thicknesses. Both gases are equally efficient, but at different temperatures [33]. The H_2_-based reduction process for the native Cu oxides is described by [34]

(1) $$Cu_{2}O+H_{2}⟶2Cu+H_{2}O$$

(2) $$CuO+H_{2}⟶Cu+H_{2}O,$$

where Cu_2_O is a native oxide, formed as a result of short exposure to ambient air. CuO was additionally formed after an exposure time of a day [35]. Figure 4a shows a temperature profile for the H_2_-assisted reduction. After sample placement on the heating plate, ambient gases were removed using vacuum steps with interim inert gas filling (N_2_) before filling the chamber with hydrogen. A reduction temperature range between 270 and 340 °C is required, depending on the sample state. The cooling process started after the reaction time of 350 s (plateau). The chamber door can be opened when the plate temperature reaches about 60 °C.

The temperature profile for the formic-acid-based reduction process is shown as the upper curve in Figure 4b. This reduction process is already very efficient at lower temperatures, and the reaction took place as follows [16]:

(3) $$Cu_{2}O+4\mathrm{HCOOH}⟶2\mathrm{Cu}{\left(\mathrm{HCOO}\right)}_{2}+H_{2}O+H_{2}$$

(4) $$\mathrm{Cu}O+2\mathrm{HCOOH}⟶\mathrm{Cu}{\left(\mathrm{HCOO}\right)}_{2}+H_{2}O$$

(5) $$Cu{\left(\mathrm{HCOO}\right)}_{2}\overset{\geq 140{}^{\circ}C}{⟶}Cu+H_{2}+2CO_{2},$$

where HCOOH is formic acid and Cu(HCOO)_2_ is Cu formate. At ambient conditions, Cu formate is formed, being temporarily stable below 140 °C, and starts decomposing at about 140 °C [16]. At 200 °C, the reduction reaction is completed by desorbing CO_2_ and H_2_, leaving the pure Cu metal. Using an integrated formic-acid module in the flip-chip bonder, tin oxides are effectively removed from the bump surface prior to the tacking process [27].

After oxide removal, the copper pads need to be protected by a Cu-formate layer using formic acid adsorption (Equations (3) and (4)). In order to initiate the reaction and produce this intermediate product, the chips are exposed to ambient conditions for at least an hour to create some native oxide. According to [15], an oxide thickness of slightly below one nanometer can be expected after an hour at room temperature. The temperature profile for formate creation is shown as the 100 °C curve in Figure 4b. As already mentioned above, the oven is equipped with an Ar/H_2_ (40/60 Vol.%) plasma source. Plasma activation was applied to test the solder wetting performance for comparison reasons. (cf. Section 3.1).

#### 2.2.2. Ball Placement

The next step concerns the solder deposition using the solder-ball bumper. Its working principle is to selectively drop each individual ball into a capillary by using a singulation disk and applying a constant nitrogen pressure through a disk hole. The solder sphere is stopped in the capillary hole, followed by activation of the laser pulse to melt the stopped solder ball. The molten ball is finally directed to wet the targeted pad through the nitrogen pressure. A detailed process description limited to 40 µm solder balls can be found in our previous paper [27]. For this work, the solder-ball bumper was equipped with a new bond head capable of using 30 µm balls, and optimization of the laser energy for the individual samples was performed. The required laser energy depends on the ball size and the pitch, and the properties of the pad material. Although solder wetting is spatially limited by the pad size, the energy stored in the molten spheres can cause solder bridging at smaller pitches. Therefore, the parameters have to be adapted to the minimal energy required to melt the balls. An energy of 0.3 mJ is needed for 30 µm balls at 50 µm pitch. A further decrease in the energy causes a capillary blockage due to insufficient solder melting. Note that 40 µm balls require 0.95 mJ for smooth running of the automated bumping process. For copper-pillar samples, 21 mJ was finally required due to the higher thermal mass of the pillars. Smaller energies are immediately consumed by the large thermal mass, thereby solidifying the solder before wetting happens. Energy exceeding 21 mJ could further improve the wetting, but at the cost of capillary burning.

#### 2.2.3. Ball Reflow

**Figure 5:**
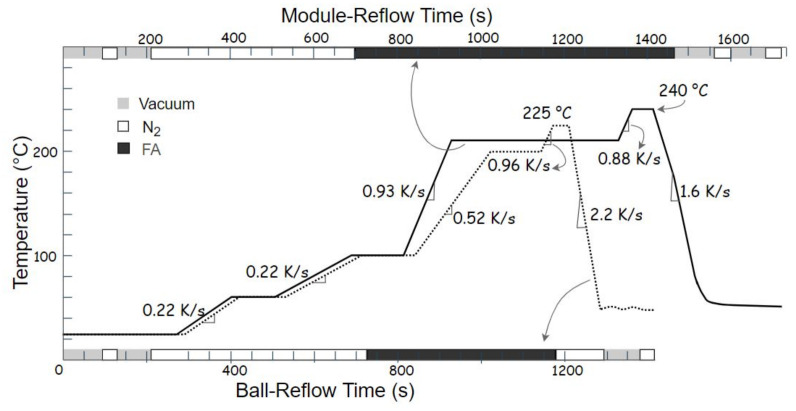
Temperature profile for the ball reflow (dotted curve) and module reflow (solid curve).

The first reflow step is performed after bumping to obtain a homogeneous bump-height profile. The ball-reflow profile shown as a dotted curve in Figure 5 was used for all the samples. The sample was placed in the vacuum oven, and an inert nitrogen atmosphere was created in two steps. The oven temperature was ramped up in steps at the rate of 0.22 K/s or 0.52 K/s during the pre-heating phase. At 100 °C, the chamber was filled with formic acid vapor. The sample was then soaked at 200 °C for 115 s to homogenize the temperature across the chip and to activate reactions between solder and copper surface. Surface oxides of tin were reduced at this stage as well. Afterwards, the temperature was increased to 225 °C at a rate of 0.96 K/s to reflow the solder bumps for about 45 s. At the end of the process, the substrate was cooled down to room temperature, and subsequent nitrogen and vacuum steps removed the formic acid vapors from the chamber.

#### 2.2.4. Tacking/Bonding

**Figure 6:**
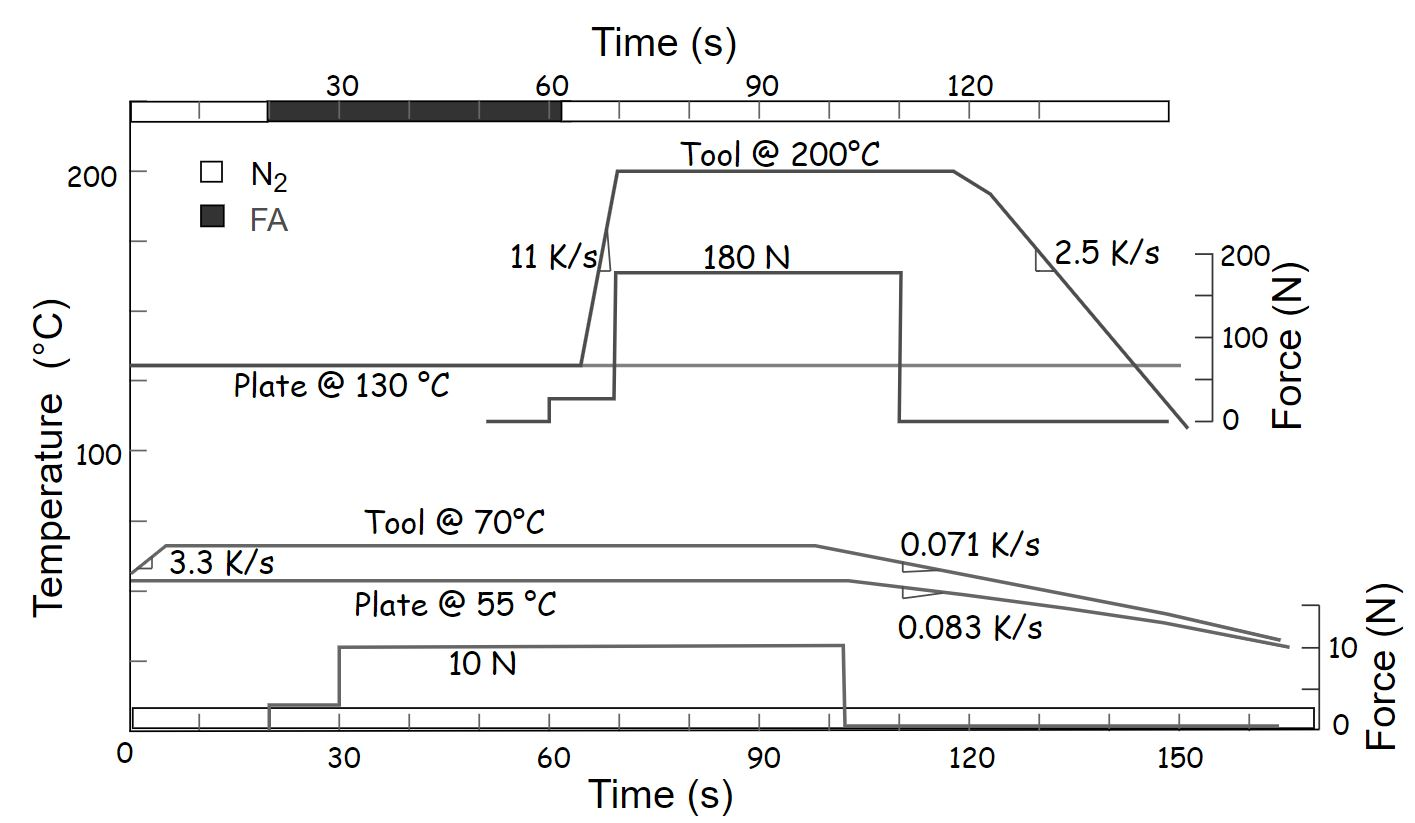
Force and temperature profiles for low-force (lower curve set) and medium-force bonding (upper curve set).

In the next step, the chips are moved into the flip-chip bonder for tacking or thermo-compression bonding. Two types of tacking profiles, i.e., low force and medium force, were used, as shown by the lower and upper curves in Figure 6. In case of the PLT module, for example, the bumped sensor Cu30/100 was placed on the heating plate which was set to a constant temperature of 130 °C. Its counterpart, the readout chip ENEPIG28, was picked up by the bond tool, which was also preset to 130 °C. This helped to reduce the thermal gradient between the bonding parts during the alignment and bonding procedure. After pad alignment, the bond tool was moved down to a distance of approximately 1 mm between the parts. At this point, formic-acid gas was flushed through the chamber flowing between the chips to maintain oxide-free bond surfaces. The readout chip was slightly touched down with 3 N (0.72 mN/bump) and leveled via a gimbal function of the tool (cf. [27] for details on gimbal leveling). The force was increased to 30 N (7.2 mN/bump) for about 10 s to ensure planarity. The flow of formic gas was stopped after the mating surfaces were in contact with each other. Finally, the tool temperature was ramped up to 200 °C to soften the solder balls. Meanwhile, the force was ramped up to 180 N (43 mN/bump: 61 MPa) and held for 40 s. At this stage, all the bumps were homogeneously deformed, and both chips were attached. The chamber was continuously flushed with inert nitrogen until completion of the bonding process. This profile was also utilized for ENIG30-ENIG30 modules but with an optimized peak force of 5 N (19.5 mN/bump: 28 MPa).

Low-temperature and low-force tacking were generally applied to all the modules with at least one Cu-based sample (except for PLT samples) and to large modules with Ni-based UBM showing pronounced warpage (e.g., ENIG80 samples). This process involved a temporary tacking material called bibenzyl. It is solid below 50 °C, liquid above this temperature and vaporizes residue-free at about 240 °C during the heating period of reflow process [36]. The lower set of curves in Figure 6 shows the temperature and force profiles of this process. Ideally, the chip without balls is placed on the heating plate, and its bumped counterpart is picked up by the bond tool. Bibenzyl crystals are carefully poured in the middle of the chip on the heating plate. Since the purpose is only temporary fixation of the aligned mating parts, about 20–30% of the chip’s surface is sufficient to hold both chips together. The temperature of the heating plate is raised above 50 °C to melt the crystals. At this point, the bond pads of mating chips are perfectly aligned with each other by the bonder’s vision alignment system. The bond head is moved down to the chip on the heating plate at a force of 3 N for 10 s. The force is then increased to a maximum of 10 N for about 70 s (156 mN/bump for Cu120: 14 MPa; 78 mN/bump for Cu80: 16 MPa; and 2.44 mN/bump for ENIG80-C4ROC/Cu80-C4ROC: 0.5 MPa). Finally, the heating plate is cooled down to resolidify the tacking medium.

#### 2.2.5. Module Reflow

At the end of the process flow, the bonded module is transferred to the vacuum oven for a final module reflow. A corresponding reflow profile is shown for the PLT module as a solid curve in Figure 5. The oven is first subjected to alternate cycles of vacuum and nitrogen to ensure a controlled reflow atmosphere. In a pre-heating phase, the plate temperature is ramped up stepwise at the rate of 0.22 K/s or 0.93 K/s. The sample is held at 210 °C for 400 s to minimize the thermal gradient across the chip at copper interface and solder material. A dwell time of 50 s above SAC305-melting point (217 °C) was chosen, with peak temperature of 240 °C for the IMC formation in the solder joint. Finally, the sample is cooled down at 1.6 K/s followed by subsequent vacuum and nitrogen steps to remove the formic acid vapors from the chamber. The peak temperature and dwell time must be adapted to the copper volume. For Cu120 and Cu80 samples, a peak reflow temperature of 270 °C and a dwell time of about 22 min were chosen to achieve a full IMC joint. Here it is worth noting that the Cu80-Cu80 samples were also reliably bonded without any ball reflow step. Such bonded module had even better solder wetting, and the full IMC point was reached in about 13 min of dwell time at 270 °C.

Table 1 summarizes the main process parameters for all the bonded samples. The tacking temperature of 55 °C corresponds to the low force bonding using bibenzyl. All the copper samples went through an initial surface cleaning using H_2_ and format protection step. The modules Cu80-Cu80 and Cu120-Cu120 gave a full IMC joint.

### 2.3. Methods of Analysis

Distinct solder wetting and high bump-shear strength are mandatory for optimum joint performance. The samples were optically inspected to assess the pad wettings. Ball-shear tests at 6 µm ball height (from the pad) were performed at two different velocities (30 µm/s, 50 µm/s) to quantify the robustness of the joints. Cross-sections were prepared by embedding the samples into epoxy and polishing them to reveal a solder-joint row. These samples were used to examine the interfacial morphologies of the joints and shape of the IMCs. A detailed analysis of the joint morphology was carried out using a scanning electron microscope (SEM) at the DESY Nanolab [37]. The chemical composition of the IMC was identified by the energy-dispersive X-ray spectrometer (EDS) of an SEM. These measurements were performed at 5 kV and 10 kV. The growth rates of different phases were estimated using the mean value of IMC thickness. The IMC thickness is calculated by dividing IMC area with IMC length along the UBM–IMC interface in cross-sectional micrographs. Functionality tests of fifteen assembled PLT modules (ENEPIG28-SAC-Cu30/100 samples) were executed to verify the connectivity yield (4160 interconnects per module) and full electrical module performance. Temperature cycling tests (TCT, 150 cycles between −40 and 125 °C) at the heating/cooling rate of 2 °C/min were performed. The dwell time at 125 °C was 20 min. TCT and high-temperature storage (HTS, 150 °C for 500 h) tests were applied to PLT modules and Cu30/100-SAC-Cu30/100 samples to monitor the mechanical robustness of the solder joints.

## 3. Results and Discussion

### 3.1. Wetting Performance

In order to find optimum conditions for solder jetting on Cu pads, two pre-studies were performed. The first study dealt with appropriate surface conditions to adsorb formic acid, and the second examined a suitable time-frame to successfully jet the solder balls on Cu formate. For this study, Cu80 samples and 40 µm solder balls were used. The samples were initially protected from oxidation and pollutions by a photoresist. After the removal of the photoresist and optical inspection, the samples were moved to the solder bumping machine. The solder jetting showed no wetting, as is evident from the microphotograph in the first column of Table 2. Assuming the pre-existence of surface impurities during or before photoresist deposition, one sample was treated with hydrogen to assure an oxide-free surface, and another sample was activated by Ar/H_2_ (40/60) plasma prior to the solder jetting. In both cases, the balls were jetted relatively centered on the pad, resulting ion improved placement accuracy, but again, without any wetting or adherence signs. Note, that these two additional samples are not included in Table 2. We assumed from these results that the unavoidable ambient exposure during sample transport from oven to solder-ball bumper caused oxide growth, thus creating a barrier between the solder and the Cu surface.

In the next step, the surface was protected using Cu formate via formic acid adsorption in the flip-chip bonder. The jetting result is shown in the second column of Table 2 (cf. FC Bonder). The semi-closed chamber led to high oxygen content, preventing the stochiometric ratio HCOOH:O of 2:1 necessary to form Cu formate [38]. That affirmed the necessity of a vacuum to perform the controlled chemical reactions. The third column presents results for when the copper surface was first activated by plasma and then exposed to ambient condition for about 15 to 30 min to induce oxide-layer growth before formate production in the oven. Taking the above-mentioned empirical relationship into account, a native oxide thickness of about 0.7 nm can be estimated for an exposure time of about 30 min [15]. The solder jetting was successful with a shear force of 14 gf. However, the solder did not optimally wet the copper pad. This process was repeated with hydrogen reduction instead of plasma activation (see the 4th column in Table 2). A shear force of ∼9 gf was obtained, alongside better solder wetting. The best solder wetting and shear results ((23.09 ± 2.83) gf: (∼ 60 ± 7) MPa) were obtained for the chips, which did not see any reduction step after photoresist removal, but only formic acid adsorption in the oven. These results indicate that certain exposure to ambient air for oxide-layer growth is mandatory to reach adequate formate-layer thickness for the highest shear strengths and good wetting. Nevertheless, for samples with Cu surfaces, which were exposed to ambient conditions for much longer, a first reduction step, followed by a controlled ambient exposure of few hours, were essential before performing the Cu–formate bonding process.

**Figure 7:**
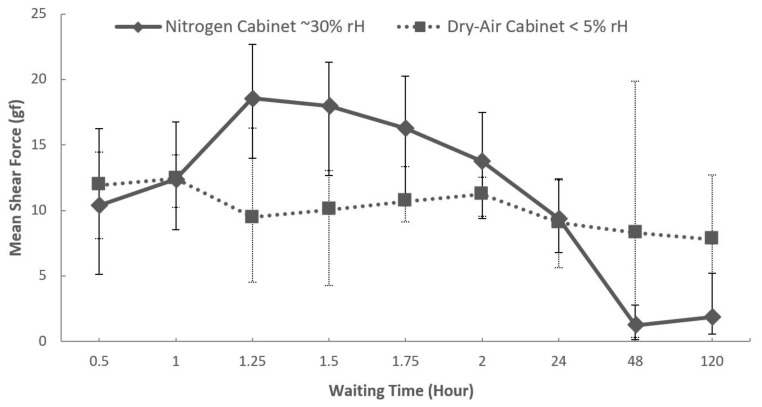
Shear force versus storage time for the samples stored in a nitrogen cabinet (solid curve) and in a dry-air cabinet (dashed curve). Each point presents a mean of at least 60 sheared balls from four different samples.

Since Cu formate is hydrophilic, the second study investigated the impacts of chip-storage conditions and time on the degradation of Cu-formate layer. Xie et al. used Cu formate to protect surfaces from oxidation during chip handling in a production line for Cu–Cu bonding, and reported its stability for a few hours [16]. We investigated the lifetime of Cu formate by storing the chips for different durations in nitrogen and dry-air cabinets. Figure 7 summarizes the life-time results obtained from measured ball-shear force versus storage time. The storage time includes the handling time of the chips under ambient conditions, i.e., microscopy and transport from oven to the designated cabinets. We observed a crucial role of humidity in dissolving the formate over time, despite storage under a nitrogen environment. A possible explanation could be the increase in oxidation rate under humid conditions. Thus, after 24 h, the native oxide grew thick enough to prevent further solder wetting. Dry-cabinet storage appeared less critical, thereby indicating that the consumption of the formate layer under exposure of dry oxygen is a slow process. Therefore, the lifetime of Cu formate in a dry air atmosphere is much longer than in a humid nitrogen atmosphere. However, in comparison to the dry-air condition, storage in nitrogen leads to higher shear strengths if the storage time can be limited up to about two hours. For this reason, all further bonding attempts were based on the samples being temporarily stored in nitrogen.

### 3.2. Bonding at 50 µm Pitch

**Figure 8:**
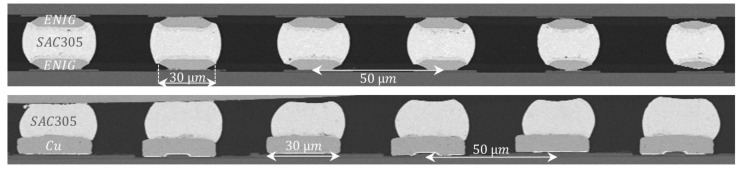
Cross-sectional view of ENIG30-SAC-ENIG30 bonded module (**top**) and a bumped Cu30/50 sample (**bottom**), both at 50 µm pitch.

Figure 8 (top) displays a cross-sectional view of a bonded ENIG30 module and demonstrates through an example the feasibility of 50 µm pitch when using 30 µm balls.

Neither interface failures nor micro-cracks were found, but approximately 30% of all inspected joints showed voids within the Sn matrix with a maximum volume of ∼4% of the solder volume. We appraise this value as uncritical as per void classification in IPC-7095 standard [39]. The lower row presents a bumped Cu30/50 sample before ball reflow, showing no bridging defects or apparent interface failures.

### 3.3. Intermetallics

#### 3.3.1. Initial IMCs

**Figure 9:**
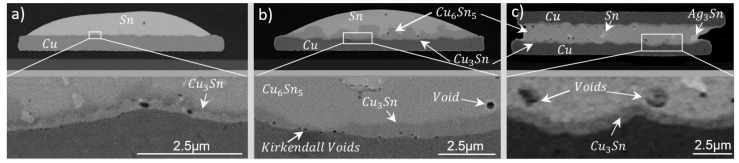
Cu80 sample with a 40 µm solder ball on a 80 µm pad after bumping (**a**) and ball reflow; (**b**) full IMC joint after tacking and module reflow (**c**). The second row presents a zoomed view of the marked rectangular area at the pad interface.

**Figure 10:**
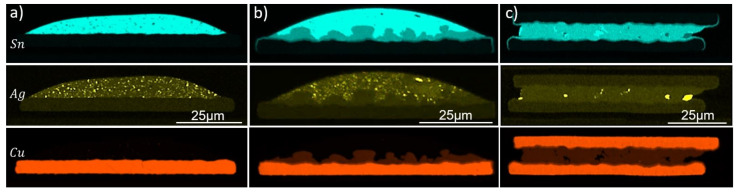
Sn (**top**), Ag (**middle**) and Cu (**bottom**) map after bumping (**a**), ball reflow (**b**) and module reflow (**c**).

Initial IMCs refer to the phases grown during the assembly step. SEM images of Cu80 samples at three stages of the bonding process are shown in Figure 9. Corresponding elemental maps are summarized in Figure 10. From the Cu–Sn phase diagram, it can be seen that two possible phases are formed with our process parameters. These are Cu_6_Sn_5_ and Cu_3_Sn, which have melting temperatures of 415 and 640 °C, respectively. We shall discuss the formation of these and other possible phases in the Cu-SAC-Cu joint. In the left picture of Figure 9, after bumping, copper was clearly completely wet by the solder. At this point, a fast growing copper-rich IMC was already formed. This IMC had a composition of 53–68 wt.% Cu and 32–46 wt.% Sn and was most probably Cu_3_Sn. Its thickness across the SAC-Cu interface was on average about 300 nm. Ag was homogeneously distributed as dot-like structures within the tin volume (cf. Figure 10a).

During the ball reflow (Figure 9b), a Sn-rich intermetallic Cu_6_Sn_5_ is formed, caused by the diffusion of Cu between the Cu-rich intermetallic region and Sn. During the final tacking and reflow step (Figure 9c), Cu_6_Sn_5_ grows and almost completely consumes the residual Sn through a solid–liquid diffusion reaction. Cu_3_Sn is present on both Cu interfaces. Over longer bonding times, Cu_3_Sn would continue growing until it completely consumes Cu_6_Sn_5_ to form a single Cu_3_Sn phase [40]. This reaction is solid–solid diffusion, since the melting temperature of both interacting IMCs is far above the reflow temperature (270 °C). With our process parameters, a very small residual amount of Sn could be seen, along with some Ag-based structures. Ag was segregated at specific points as a Ag_3_Sn phase, as shown in the Ag map (cf. Figure 10c). The IMC growth rate of dominant phases is given by

(6) $$d_{0}=k\sqrt{t}$$

where d_0_ is the thickness of the IMC after the final assembly step, k is the growth constant and t is the dwell time. Since the Sn consumption was almost completed, we estimated Cu_6_Sn_5_ and Cu_3_Sn growth-rate constants of about 140 nm/$\sqrt{s}$ and 29 nm/$\sqrt{s}$, respectively. These values are in reasonable agreement with [41], wherein a growth-rate constant of 161.3 nm/$\sqrt{s}$ (Cu_6_Sn_5_ + Cu_3_Sn) was obtained for a bonding temperature of 325 °C. From this, one can estimate a full IMC growth time of about 5 min when using 30 µm balls instead. A few small voids in the Cu_6_Sn_5_ layer and Kirkendal voids in Cu_3_Sn layer were formed. A former study investigated the void-formation mechanism in Cu-Sn-Cu system and reported similar voids in Cu_6_Sn_5_ near the Cu_3_Sn layer [42]. Their parameters led to large porosity in Cu_6_Sn_5_ after full-phase formation. However, our parameters lead to only a few nano-voids and no cracks in the final full-phase joints. Note, that these samples had a thick polymer coating covering the edges of the round copper pads to prevent solder creep from pads to the joint copper traces. The Sn map of the bonded sample in Figure 10c clearly shows that the sandwiched solder did not spread to the edges but was confined by the passivation. However, it can be seen that a very small amount of tin flowed underneath the passivation to the edges of the copper pads.

**Figure 11:**
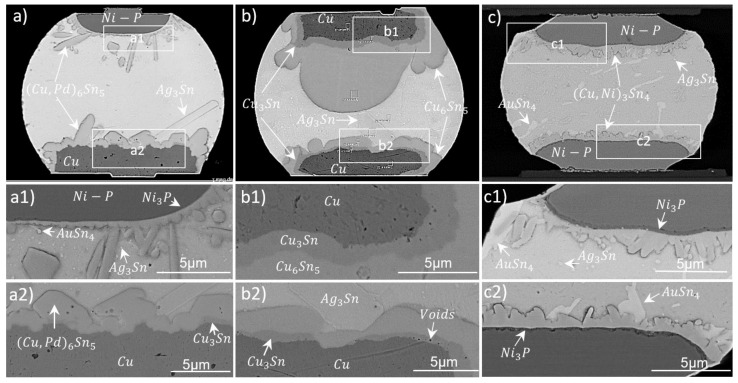
Cross-sections from the 30 µm bump-bond process showing an ENEPIG28-SAC-Cu30/100 (PLT) (**a**), a Cu30/100-SAC-Cu30/100 (**b**) and an ENIG30-SAC-ENIG30 (**c**) solder joint. Magnified views of upper and lower UBM interfaces for three samples are shown as (**a1**), (**b1**) and (**c1**); and (**a2**), (**b2**) and (**c2**), respectively.

Figure 11 shows cross-sections of three different bonded samples with narrow pads utilizing 30 µm bumps. Figure 11a originates from a PLT module, where the solder balls were placed on the sensor side (lower Cu contact) on Cu30/100. The dominant phases at both Cu-SAC and ENEPIG-SAC interfaces were Cu-Sn based, with a little amount of Pd. The ternary phase consisted of 25–40 wt.% Cu, 51–62 wt.% Sn and 1–3 wt.% Pd and is known as (Cu, Pd)_6_Sn_5_. This phase was more flattened and intact on the Cu side, but it was more needle-like and spalled off the interface on Ni side. Ni-based phases were not observed at the ENEPIG–SAC interface.

A possible reason could be a larger diffusion coefficient of Cu atoms; thus, Cu from opposite interface would diffuse rapidly during the liquid phase of the solder to form (Cu, Pd)_6_Sn_5_ phases at the ENEPIG–solder interface. Additionally, a ∼130 nm (thin) Ni_3_P layer between Ni and (Cu, Pd)_6_Sn_5_ (see Figure 11(a1)) was formed, both acting as barrier layers for Ni. The growth constant is estimated to be ∼13 nm/$\sqrt{s}$ from this thickness of Ni_3_P. For the upper interface, the mean thickness and growth-rate constant of the (Cu, Pd)_6_Sn_5_ IMC were about 2 µm and 195 nm/$\sqrt{s}$, respectively. For the lower interface, the mean thickness and growth-rate constant of the (Cu, Pd)_6_Sn_5_ IMC were about 3.6 µm and 365 nm/$\sqrt{s}$, respectively. Plate-like structures of Ag_3_Sn (69–72 wt.% Ag, 27–31 wt.% Sn) were present near the Cu interface [43]. Ag_3_Sn was also uniformly present as dot-like structures within the bulk Sn. Some dot-like AuSn_4_ structures were also spotted. Cu_3_Sn grew partially at the interface after the final bonding process (see Figure 11(a2)). Very rare nanovoids were spotted in the IMC at the Cu interface. We discuss the voiding in this sample after the aging experiment in Section 3.3.2.

Figure 11b shows a Cu30/100-SAC-Cu30/100 solder joint, and micrographs in Figure 11(b1,b2) show zoomed views of both copper–solder interfaces. At both interfaces, a Sn-rich Cu_6_Sn_5_ layer was formed, followed by a Cu-rich Cu_3_Sn layer. The thicknesses (growth-rate constant) of the Cu_3_Sn layer were about 1.5 µm (41 nm/$\sqrt{s}$) and 800 nm (22 nm/$\sqrt{s}$) at the upper and lower interface, respectively. The mean thicknesses (growth-rate constant) of Cu_6_Sn_5_ were about 10 µm (295 nm/$\sqrt{s}$) and 3 µm (82 nm/$\sqrt{s}$) at the upper and lower interfaces, respectively. The pronounced IMC growth on the upper Cu-SAC interface was due to two reflows (ball reflow at 225 °C for 40 s and module reflow at 270 °C for 1320 s). The IMC thickness derived after ball reflow process was about 1 µm corresponding to a growth-rate constant of 172 nm/$\sqrt{s}$, which shows that major growth on the upper interface happens during the module reflow process. When comparing it with the opposite interface, it can also be observed that the IMC growth during module reflow was much enhanced through an already existing phase (due to ball reflow) on the upper interface. Additionally, Cu_3_Sn was more enhanced and homogeneous on the upper side. The growth of the IMC can be controlled by changing the dwell time and peak temperature in the final reflow step. An increase in dwell time can lead to a full IMC joint. From the growth-rate constants mentioned above, a dwell time of about 30–40 min should lead to full IMC formation. As in other samples, Ag from the SAC segregates near the interface, forming an elongated, plate-like Ag_3_Sn phase.

Figure 11c presents a magnified image of a solder joint from the ENIG30-SAC-ENIG30 module, which is shown in the upper row of Figure 8. Figure 11(c1,c2) present magnified views of upper and lower interfaces, respectively. EDS studies detect mainly (Cu,Ni)_3_Sn_4_ phases with the composition of 31 wt.% Ni, 65 wt.% Sn and 4 wt.% Cu [44]. At the upper interface, the mean thickness and growth-rate constant of the (Cu,Ni)_3_Sn_4_ phase were about 1.2 µm and 139 nm/$\sqrt{s}$, respectively. For the lower interface facing two reflows, the mean thickness and growth-rate constant of the (Cu,Ni)_3_Sn_4_ phase were about 1.55 µm and 179 nm/$\sqrt{s}$, respectively. Au and Ag were distributed in the Sn matrix in the forms of AuSn_4_ (long whitish structures: 70 wt.% Sn, 30 wt.% Au) and Ag_3_Sn (small gray dots: 50–70 wt.% Sn, 29–48 wt.% Ag) structures. A 100 nm (thin) layer of Ni_3_P (12 nm/$\sqrt{s}$) appeared at the interfaces, preventing further Ni consumption into the solder. All IMCs are characteristic for this type of UBM, as discussed in our previous study [27]. Compared to the sample with a Cu-based UBM of similar dimensions and similar reflow parameters (Figure 11a), the IMCs at the UBM-solder interfaces were more pronounced and had higher growth-rate constants in the Cu sample. IMC growth is more pronounced when both mating chips are Cu-based (Figure 11b). This sample is a good candidate for producing a full phase joint for stacking applications. None of the three samples showed any void failure or interface defects.

**Figure 12:**
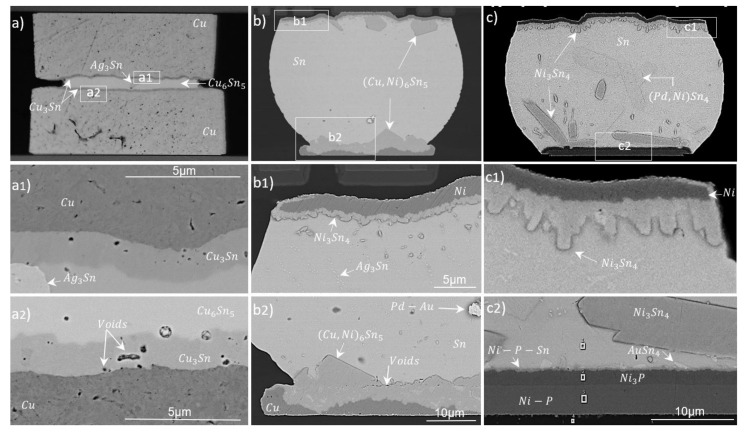
Solder-joint cross-sections of a bonded Cu120-SAC-Cu120 (double pillar) sample with a 40 µm solder ball (**a**); Cu80-C4ROC (**b**) and ENIG80-C4ROC samples with 100 µm C4 solder bump (**c**). Magnified views of upper and lower UBM interfaces for three samples are shown as (**a1**), (**b1**) and (**c1**); and (**a2**), (**b2**) and (**c2**), respectively.

The cross-sections in Figure 12 present pillars and large-format chips bonded by the low-force technique. Figure 12a shows two Cu120 samples bonded with a 40 µm ball (pillar on pillar). The bonded module had a full IMC joint due to the small volume of SAC interacting with copper. Ag from the SAC aggregated at specific points and formed Ag_3_Sn phases. The Cu_3_Sn layer at both Cu–SAC interfaces had a mean thickness and growth-rate constant of about 1.2 µm and 33 nm/$\sqrt{s}$, respectively. Sn from the middle joint area was almost completely consumed in Cu_6_Sn_5_ with a growth constant of about 63 nm/$\sqrt{s}$. A few Kirkendall voids were visible in Cu_3_Sn, but no cracks were spotted in the joint or at interfaces. Such a joint can be applied to produce stand-off height in a complex 3D packaging scheme.

Figure 12b presents a large-format Cu80 sample bonded with a C4ROC having 100 µm C4 SnAg bumps. The solder wetting on the Cu pad is confined by thick passivation (see beak-shaped corners). (Cu, Ni)_6_Sn_5_ phases with compositions of about 26 wt.% Cu, 16 wt.% Ni and 58 wt.% Sn were formed on both interfaces. The growth-rate constants for this phase were 189 nm/$\sqrt{s}$ and 210 nm/$\sqrt{s}$ at top (Ni-SAC) and bottom (Cu-SAC) interfaces, respectively. On the top interface, a thin Ni_3_Sn_4_ (31 wt.%Ni–69 wt.%Sn) phase was formed, right above the Ni. Compared to the Ni-based sample in (Figure 12(c2)), it can be observed that an absence of a barrier layer on the copper can lead to a maximum consumption of the UBM due to a large solder-to-UBM volume. This could raise reliability concerns for high-temperature applications.

Figure 12c shows the same C4ROC bonded with an ENIG80 sample at a peak temperature of 240 °C with a dwell time of ∼70 s. The final IMCs were clearly different as compared to the Cu-based interface. An about 1.4 µm thick Ni_3_P layer with a growth-rate constant of about 167 nm/$\sqrt{s}$ was formed at the bottom UBM interface. As a result of interaction between Ni_3_P and Sn, a ternary phase of Ni-P-Sn with an approximate thickness of 400 nm and a growth-rate constant of about 48 nm/$\sqrt{s}$ was formed above the Ni_3_P layer. These phases protected Ni from further consumption in the interfacial reactions. The most prominent phases were scallop-like Ni_3_Sn_4_ structures at the sensor side (lower interface). Their phase composition was 28–35 wt.% Ni and 65–72 wt.% Sn. A similar phase was distributed as a homogeneous layer on the top side. Some thicker rectangular-shaped light-gray structures within the bulk Sn were (Pd, Ni)Sn_4_ phases with compositions of 7–11 wt.% Ni, 7–8 wt.% Pd and 82–86 wt.% Sn [45]. Au and Ag were homogeneously distributed in the solder as tiny dot-like structures.

Table 3 summarizes the most prominent IMCs, along with their growth constants and process parameters. It can be seen that the interfacial reactions are enhanced in Cu-based UBM. This property can be used to produce full IMC joints for stacking applications. Moreover, full phase joints using various solder sizes can assist different stand-off heights in complex 3D packages.

#### 3.3.2. Post-TCT and -HTS IMCs

**Figure 13:**
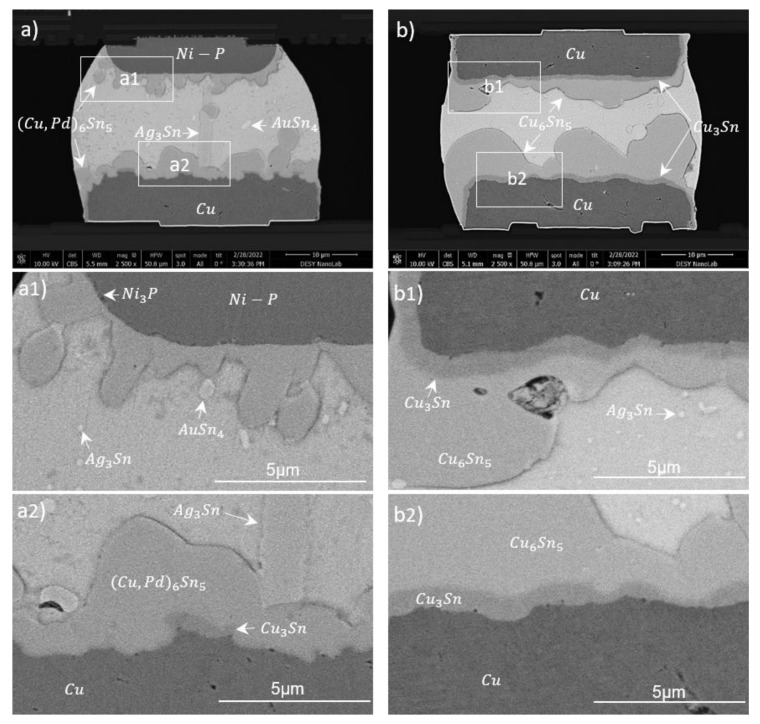
Cross-sectional view of Cu30/100-SAC-ENEPIG28 (**a**) and Cu30/100-SAC-Cu30/100 (**b**) solder joints after TC tests. Magnified views of upper and lower UBM interfaces for both samples are shown as (**a1**) and (**b1**); and (**a2**) and (**b2**), respectively.

The solder-joint cross-sections of the TC-tested Cu30/100-SAC-ENEPIG28 (PLT) and Cu30/100-SAC-Cu30/100 modules are displayed in Figure 13. They confirm crack-free robust interconnects. In comparison to the corresponding cross-sectional images in Figure 11a,b, both images attest a nearly unchanged phase front after thermo-mechanical stress loading. However, the shapes of phases on the Ni-SAC interface changed from sharp scallop-like structures to a more flattened layer covering the interface.

**Figure 14:**
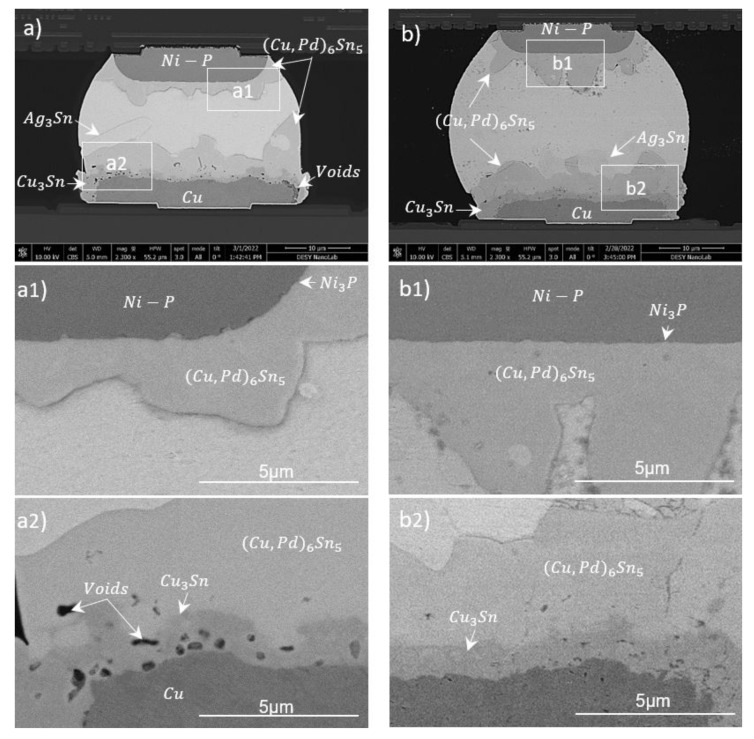
HTS tests of Cu30/100-ENEPIG28 modules bonded using 30 µm spheres (**a**) and 40 µm spheres (**b**). Magnified views of upper and lower UBM interfaces for both samples are shown as (**a1**) and (**b1**); and (**a2**) and (**b2**), respectively.

Figure 14a presents cross-sections of the Cu30/100-SAC-ENEPIG28 solder joints of the PLT modules subjected to HTS tests. The comparison of the IMC-phase fronts before (cf. Figure 11a) and after HTS depicts well-pronounced growth of (Cu, Pd)_6_Sn_5_ IMCs at the top and the bottom interface and of Cu_3_Sn IMCs on the bottom side. Since stress temperature is far below the melting temperature of the solder and IMCs, the interaction between the solder and IMC is based on solid-state diffusion of involved elements. We calculated the IMC growth rate using equation

(7) $$d=d_{0}+k\sqrt{t},$$

where d_0_ and d are the IMC thickness after assembly and after HTS, respectively. We estimated growth-rate constants of (Cu, Pd)_6_Sn_5_ IMCs of about 0.9 nm/$\sqrt{s}$ and 0.8 nm/$\sqrt{s}$ for the upper and lower interface, respectively, after 500 h at 150 °C. A prominent difference was seen on the Cu30/100-SAC interface (compared to the same sample after bonding and temperature cycling), where more copper was consumed to enhance the Cu_3_Sn layer. Meanwhile, the Cu diffusion to Cu-Sn-Pd creates vacancies leading to Kirkendall voids, as also observed by [46]. The SEM micrographs show an uneven Cu_3_Sn-layer thickness across the interface; the maximum thickness was about 1.5 µm. The growth-rate constant was about 1 nm/$\sqrt{s}$. Cu_3_Sn was not clearly visible after bonding or temperature cycling. The growth rate at room temperature is known to be extremely low [47]. A recent study investigated Cu_6_Sn_5_ + Cu_3_Sn IMC growth as a function of reflow time [48]. The reflow time of 1 min (closest to our dwell time) only produced Cu_6_Sn_5_. Cu_3_Sn appeared in the samples with higher reflow durations or could clearly be seen after 72 h of aging at 200 °C. They reported higher growth constants during aging for the samples with smaller reflow times and lower initial IMC thicknesses.

Chi et al. [49] found that Cu_3_Sn starts to appear after about 64 h of aging at 150 °C, and its thickness increases with further aging. They mainly studied the reliability aspect of ultra-thin UBM on the copper substrate by discussing resulting phases in regard to aging and drop-test performance. A former study investigated the use of barrier metals on Cu to avoid the growth of Cu_3_Sn, which is often followed by Kirkendall voids [50]. This is essential for the applications, where chips are exposed to extreme temperatures for longer periods, and hence the voids have a tendency to generate cracks, and finally complete interface failure. This is, however, not critical for our detector applications utilizing cooling and operating usually at and below room temperature.

For comparison, the Cu30/100-ENEPIG28 module using 40 µm spheres was also temperature stressed and is shown in Figure 14b. Preliminary results showed an increase in void occurrence for lower solder volumes, which is visible in magnified images in Figure 14(a2,b2). Nevertheless, the voids in 30 µm joints did not initiate any cracks or joint failure.

### 3.4. Shear Strength

In this section, we compare the shear results of Cu30/100 samples using 30 µm solder spheres with our previous results of 40 µm solder spheres on NiPdAu UBM [27]. Both samples were sheared at a height of 6 µm (from UBM–solder interface to the solder direction) at a speed of 30 µm/s. This shear height corresponds to about 20% and 15% of the 30 µm and 40 µm ball heights, respectively. Further process steps after ball placement enhanced the IMCs, and thus higher shear forces were obtained for the same shear height. Shear height’s dependency on the shear strength was also studied for the NiPdAu samples, showing a decrease in shear strength with increasing shear height (moving away from the interface with IMCs) [27].

**Figure 15:**
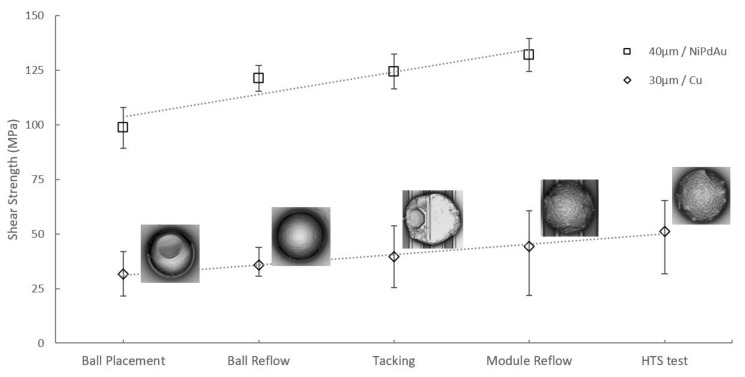
Comparison of shear strengths between Cu30/100 with 30 µm solder spheres and NiPdAu-UBM samples with 40 µm solder spheres. Each point presents a mean of at least 100 sheared balls from four different samples.

Figure 15 shows that the shear strengths for copper-based UBM were about a factor of four smaller than for NiPdAu UBM, but were still well above the Mil-Std 883H standard of 10 MPa [51]. Since the Kirkendall voids did not induce a crack on the interface, the shear strength increased with increasing IMC thickness after HTS tests.

### 3.5. Electrical Test

Altogether, sixteen PLT modules were assembled for electrical and performance qualification. Except for the cross-sectional sample, all of them were finally tested, covering the full module performance. All modules passed the qualification with less than 1% defect pixel. Three modules showed minor defects, one had two defective pixels, one had one defective pixel and two missing interconnects and one had one missing interconnect. Due to these excellent test results, the PLT modules were finally mounted on the CMS experiment [28]. Full functionality tests for the corresponding NiPdAu UBM modules are discussed in [27]. Both results confirm the electrical appropriateness of the introduced in-house processes.

## 4. Summary and Conclusions

This paper described a successful application of an in-house bump-bond and solder reflow process for chip-to-chip interconnects on various samples differing in chip size, pad size, pitch, pad material and storage conditions. Various UBM combinations of mating chips (Cu-SAC-Cu, ENEPIG-SAC-Cu, ENIG-SAC-ENIG) were reliably bonded using different solder volumes. The main challenge of direct solder bumping on the copper pads and pillars under ambient conditions was addressed. Prior to the solder bumping, process gases were used to eliminate native oxide from the copper surfaces, followed by building a Cu–formate layer for surface protection and solder wetting. Cu80 samples were utilized to investigate the lifetime of Cu–formate in nitrogen and dry-air storage environments to employ a suitable time window for optimal solder bumping. The process was successfully demonstrated for placement of 30 µm solder balls at 50 µm pitch on both Cu and NiAu UBM. The low-force and low-temperature bonding technique was reliably applied to large-format Cu- and NiAu-based samples. Intermetallic compounds in all the samples were characterized through EDS, and their growth-rate constants were estimated. Cu80 and Cu30/100 samples were used to asses the joint reliability through shear tests. PLT modules consisting of Cu30/100 and ENEPIG28 samples were subjected to thermal stress and high-temperature storage tests, demonstrating the robustness of the underlying processes.

As concluding remarks, we can say that the flexibility offered in sizes and materials makes our in-house process quite suitable for rapid prototyping. The full IMC joints provide a good basis for proof-of-concept studies for stacked packages. Based on the described study and our experimental results, we propose a protocol for a reliable bonding of Cu-based samples for applications at room temperature and below. To ensure reliability for high temperature applications, we suggest deposition of a NiAu film on top of copper. 

## Figures and Tables

**Table 1:** Summary of the process parameters, including ball size, peak temperature (T), dwell time (t) and bonding force (F), for all the bonded samples.

Bonded Samples	Ball Size	Ball Reflow	Tacking		Module Reflow
	µm	T (°C), t (s)	T (°C)	F (MPa)	T (°C), t (s)
Cu80-Cu80	40	225, 45	55	16	270, 1320
ENEPIG28-Cu30/100	30	225, 45	180	61	240, 100
Cu30/100-Cu30/100	30	225, 45	55	3.4	270, 1320
ENIG30-ENIG30	30	225, 45	180	28	240, 75
Cu120-Cu120	40	225, 45	55	14	270, 1320
C4ROC-Cu80	100	225, 45	55	0.5	240, 500
C4ROC-ENIG80	100	225, 45	55	0.5	240, 70

**Table 2:** Wetting performance and shear results for 40 µm solder balls on Cu80 samples after various surface treatments. The balls were sheared at a height of 6 µm from the bond pad with a speed of 50 µm/s.

		FC Bonder	Vacuum Oven		
	Photoresist Removal	Cu Formate	Ar/H_2_ Plasma + Cu Formate	H_2_ + Cu Formate	Cu Formate
Shear Force (gf)	0	0.43	14.09	9.23	23.09
Standard Deviation (gf)	0	0.26	5.66	4.4	2.83
Wetting on Cu80	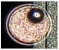	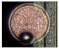	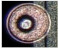	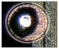	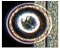

**Table 3:** Summary of the interfacial IMCs with peak temperatures (T), dwell times (t), thicknesses (d_0_) and growth-rate constants (k) after the final assembly step. (t) stands for top interface and (b) for bottom UBM-solder interface, as shown in Figure 11 and Figure 12.

Bonded Sample	T	t	Type	d_0_	k
(t)-(b)	°C	s		µm	nm/$\sqrt{s}$
Cu80-Cu80	270	780	Cu_3_Sn	0.8	29
			Cu_6_Sn_5_	3.9	140
ENEPIG28-Cu30/100	240	100	Ni_3_P	0.13	13 (t)
			Cu-Pd-Sn	1.95 (t), 3.65 (b)	195 (t), 365 (b)
Cu30/100-Cu30/100	270	1320	Cu_3_Sn	1.5 (t), 0.8 (b)	41 (t), 22 (b)
			Cu_6_Sn_5_	10.7 (t), 2.99 (b)	295 (t), 82 (b)
ENIG30-ENIG30	240	75	Ni_3_P	0.1	12
			(Cu, Ni)_6_Sn_5_	1.2 (t), 1.55 (b)	139 (t), 179 (b)
Cu120-Cu120	270	1320	Cu_3_Sn	1.18	33
			Cu_6_Sn_5_	2.29	63
C4ROC-Cu80	240	500	(Cu, Ni)_6_Sn_5_	4.2 (t), 4.7 (b)	189 (t), 210 (b)
C4ROC-ENIG80	240	70	Ni_3_P	1.4	167 (b)
			Ni-P-Sn	0.4	48 (b)
			Ni_3_Sn_4_	2.99 (t), 2.12 (b)	358 (t), 253 (b)

## Sample Availability

We are grateful to L. Andricek from the Semiconductor Laboratory of MPG for providing Cu80 and ENIG30 samples; T. Oppert from Pactech GmbH for providing Cu120 samples; and J. Schwandt from the Institute of Experimental Physics, University of Hamburg, for Cu30/50 and Cu30/100 samples. ENEPIG28 samples stemmed from the CMS BPIX upgrade project; ENIG80 and C4ROC samples were from the DSSC project.
